# Machine learning reduces soft costs for residential solar photovoltaics

**DOI:** 10.1038/s41598-023-33014-4

**Published:** 2023-05-03

**Authors:** Changgui Dong, Gregory Nemet, Xue Gao, Galen Barbose, Benjamin Sigrin, Eric O’Shaughnessy

**Affiliations:** 1 School of Public Administration and Policy, Renmin University of China, Beijing, 100872 China; 2 La Follette School of Public Affairs, University of Wisconsin-Madison, Madison, WI 53706 USA; 3 Department of Political Science, University of Miami, Coral Gables, FL 33146 USA; 4 Present Address: Askew School of Public Administration and Policy, Florida State University, Tallahassee, FL 32306 USA; 5 Lawrence Berkeley National Laboratory, Berkeley, CA 94720 USA; 6 National Renewable Energy Laboratory, Golden, CO 80401 USA; 7 Clean Kilowatts, LLC, Boulder, CO 80302 USA

**Keywords:** Climate change, Climate-change mitigation

## Abstract

Further deployment of rooftop solar photovoltaics (PV) hinges on the reduction of soft (non-hardware) costs—now larger and more resistant to reductions than hardware costs. The largest portion of these soft costs is the expenses solar companies incur to acquire new customers. In this study, we demonstrate the value of a shift from significance-based methodologies to prediction-oriented models to better identify PV adopters and reduce soft costs. We employ machine learning to predict PV adopters and non-adopters, and compare its prediction performance with logistic regression, the dominant significance-based method in technology adoption studies. Our results show that machine learning substantially enhances adoption prediction performance: The true positive rate of predicting adopters increased from 66 to 87%, and the true negative rate of predicting non-adopters increased from 75 to 88%. We attribute the enhanced performance to complex variable interactions and nonlinear effects incorporated by machine learning. With more accurate predictions, machine learning is able to reduce customer acquisition costs by 15% ($0.07/Watt) and identify new market opportunities for solar companies to expand and diversify their customer bases. Our research methods and findings provide broader implications for the adoption of similar clean energy technologies and related policy challenges such as market growth and energy inequality.

## Introduction

Deploying renewable energy technologies is key to mitigating climate change and fostering an energy transition^1–3^. Driven by rapid hardware cost reductions, solar photovoltaics (PV) is ready to be subsidy-free and power a sustainable future^4,5^. However, PV non-hardware or “soft” costs now account for over 60% of installed prices and are more resistant to reductions than hardware costs^6^. Soft cost stagnation could slow PV diffusion^7–9^. Customer acquisition costs, i.e., PV companies’ costs to identify and acquire new customers, are currently the largest component of PV soft costs. Customer acquisition costs amounted to 21% ($0.43/Watt) of total PV soft costs in the U.S. in 2020^10^. Furthermore, customer acquisition costs have been rising as rooftop solar diffusion shifts from early adopters to mass diffusion, and as customers get fatigue from door-to-door marketing, creating a substantial challenge for PV companies^10,11^. Developing efficient and effective methods to identify prospective PV adopters could reduce customer acquisition costs, accelerating technology diffusion and the associated climate benefits. Improved adoption prediction could also benefit grid infrastructure planning, transmission and storage siting, and subsidy policy design for low-income communities via adoption ‘seeding’^12^.

Predicting household PV adoption differs from explaining PV adoption. Prior research has relied on significance-based methods with an extensive focus on the differences between PV adopters and non-adopters^13–16^, factors that motivate PV adopters^17–21^, and factors that hinder non-adopters^22–24^. So far, 333 household-level factors have been associated with PV adoption in the literature^13^. Logistic regression is the most commonly used method to estimate the effects of those factors on adoption for adopters^7,25,26^, non-adopters^27,28^, or both groups^18,29–33^. Yet, there is no overarching theory on the economic, social, political, and psychological drivers of PV adoption^21,34^. Furthermore, the predictive value of those many household variables to PV adoption remains understudied. Recent advances in statistics suggest that highly significant variables are not necessarily good predictors because significance tests and predictions rely on different properties of the underlying unknown distributions^35,36^. Therefore, a shift from identifying highly significant variables to building highly predictive models may represent a new path for future works, making academic research more useful to the industry and policy-making.

Machine learning algorithms are better suited than significance-based regression methods for prediction tasks such as identifying PV adopters. Machine learning methods are well-equipped to capture complicated interactions among variables and their nonlinear patterns while penalizing model complexities^37,38^. Such capabilities contrast with the (quasi-)linearity assumed in most PV adoption studies that are regression-based. Our research uses machine learning to focus on predicting rather than explaining PV adoption. With enhanced prediction performance on households’ PV adoption behavior, our research could be used to lower customer acquisition costs and identify new market opportunities for PV installation companies. Many studies in the literature have applied machine learning methods to estimate solar potential^39,40^ and deployment density^41–43^, detect solar panels in aerial images^44–46^, and to forecast PV power generation^47–49^. Two recent studies also used machine learning to classify PV adopters and non-adopters; however, they either had very limited data on PV adopters (e.g., 30 adopters)^50^ or their prediction performance could be further improved^51^.

Our research leverages the largest survey data sample collected from both PV adopters and non-adopters in the United States (*N* = 3570; see Supplementary Table S1 for comparisons with previous studies), and employs a state-of-art machine learning algorithm (XGBoost, eXtreme Gradient Boosting) to predict household PV adoption. We first compare the predictive performance of the chosen machine learning algorithm with logistic regression, the dominant significance-based method. We also show the superior performance of XGBoost relative to other classic machine learning algorithms (e.g., support vector machine and random forest). We further dive into the modeling detail of XGBoost and decompose its enhanced prediction performance over logistic regression into two factors: variable interaction and nonlinearity. We last show the potential of XGBoost in reducing customer acquisition costs, and then the ability to identify new market opportunities for PV companies.

Overall, this study suggests a meaningful shift from significance-based models to prediction-oriented methods, and demonstrates that machine learning algorithms are able to improve the prediction performance for PV adoption, lower PV companies’ customer acquisition costs, make the technology affordable to more people, and further increased the market size of the industry. As we only use easily accessible variables in our prediction, the PV industry can easily apply our methods and results to their customer acquisition practices. Our research findings could also improve the decision-making of utility planners and policy-makers by providing a better prediction of the location and market size of future PV adoption.

## Results

### Significant variables with meager predictivity

**Figure 1:**
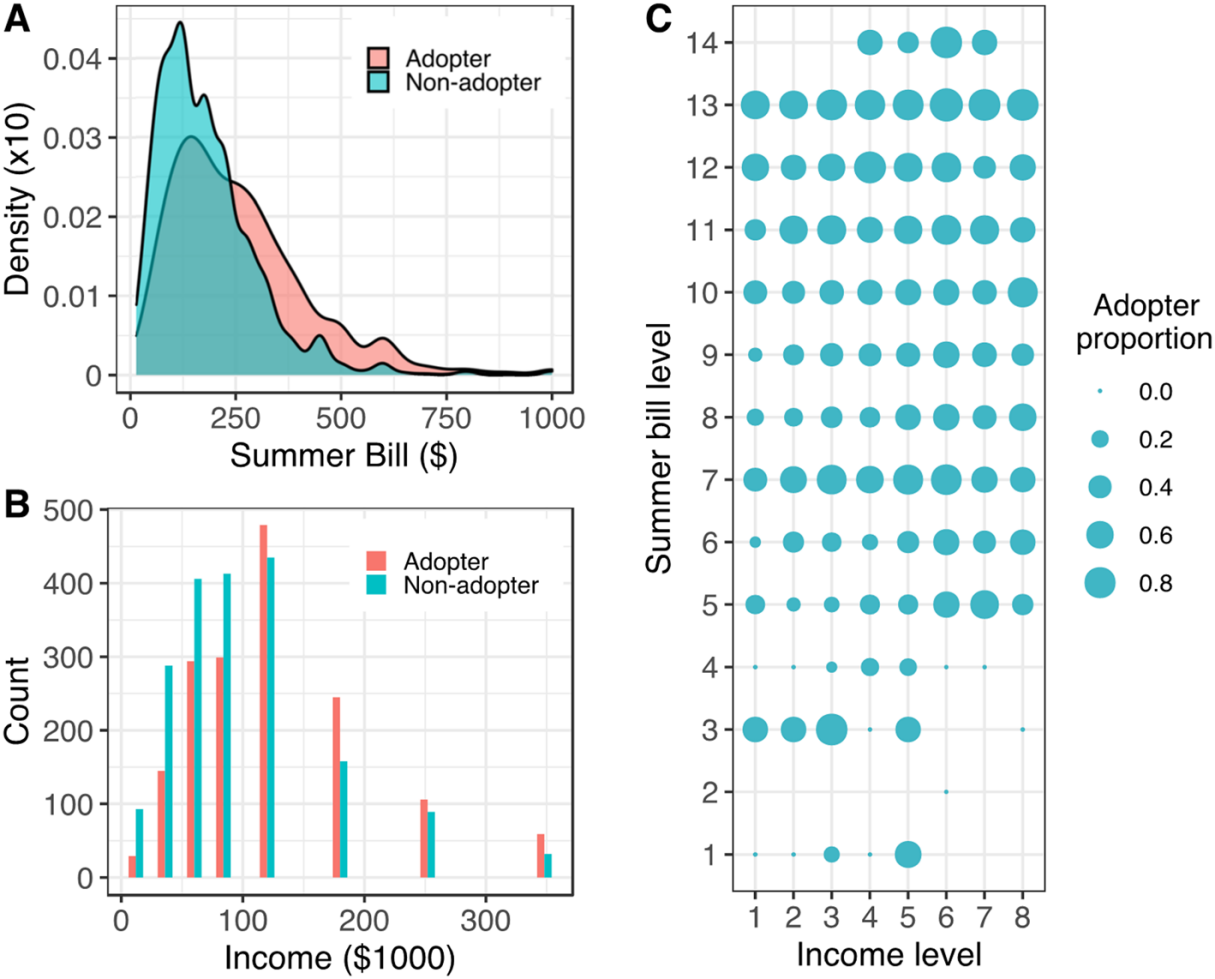
Correlations between two common household attributes and PV adoption status. (**A**) Distribution of household summer monthly bill by PV adoption status; though PV adopters have higher summer monthly electricity bills than non-adopters on average, the overlapping between these two groups is significant. (**B**) Distribution of household annual income by PV adoption status, where PV adopters have higher household income than non-adopters on average but still with significant overlaps. (**C**) Multimode patterns of PV adopter proportion with respect to household income and summer monthly bill, with summer monthly bill first discretized into 14 equal bins.

PV adopters and non-adopters differ in many household attributes (variable description and summary statistics in Supplementary Table S3). In Table 1, for example, PV adopters on average have much a higher monthly electricity bill than non-adopters, and the differences are $80 per month in summer and $40 per month in winter. Both differences are statistically significant (all $$P<0.01$$, Table 1). On household income (Fig. 1B), PV adopters have annual incomes that is $22,000 higher than non-adopters ($$P<0.01$$, Table 1). Other variables such as solar irradiation, number of household occupants, and age of household owners (Table 1) are also statistically different between adopters and non-adopters.

**Table 1:** Mean difference tests of household attributes between PV adopters and non-adopters.

Variable	Adopters	Non-adopters	Difference	Permutation test	Prediction rate
Summer bill	259.5	180.9	78.6	16.0***	0.62
Winter bill	185.5	145.6	39.9	10.3***	0.61
Capacity factor	17.3	16.3	1.0	13.6***	0.67
Income ($1,000)	121.0	99.0	22.0	9.7***	0.58
# people	2.3	2.1	0.2	7.5***	0.55
Age	57.1	53.1	4.0	8.4***	0.57

Only continuous variables are shown here (*N* = 3570); their inter-correlations are provided in Supplementary Table S5. For categorical variables, see Supplementary Table S6 for their corresponding test. Here we employ the non-parametric permutation test instead of the parametric t-test since the former does not rely on any statistical assumptions behind the data as the latter does. Block permutation test results are further provided in Supplementary Table S6 to satisfy the weaker exchangeability assumption of the observations within a block^52^.

***P < 0.01, **P < 0.05, *P < 0.10.

Differences between PV adopters and non-adopters do not automatically translate into predictivity, i.e., the highest correct prediction rate for a variable that is determined by distinguishability of the two distributions. As shown in Fig. 1A, the summer electricity bill distributions by PV adoption status substantially overlap, as does household income in Fig. 1B. Although PV adopters have a higher summer bill on average, 38% of them see a summer bill smaller than the average non-adopter’s summer bill. Following Lo et al.’s method^35^, this variable’s prediction rate (see the Methods section) is 62%. Such a prediction accuracy is only a moderate improvement over a random guess (50%), though it is one of the best predictors among all household attributes in Table 1.

Interactions between household attributes further complicate the prediction task. By interacting summer bills with household income, the proportion of PV adopters does not increase linearly in either dimension (see Fig. 1C); other measures, such as energy efficiency and different energy source options, could be influencing factors as well. A large share of PV adopters is found in all summer bill levels, and there is no obvious linear trend in the PV adopter share across income levels. These suggest that a (quasi-)linear coefficient of electricity bill or household income on the probability (or odds ratio) of adopting PV is an over-simplification. Successfully predicting who will adopt PV requires the incorporation of those nonlinear patterns and complex interactions in household attributes.

### Comparing prediction performance of logistic regression and XGBoost

Logistic regression is the most commonly used method to analyze differences between PV adopters and non-adopters^29,30,33^, serving as a good baseline to compare with machine learning algorithms. Our logistic regression model with nine original and highly visible household features successfully predicts 71% of out-of-sample PV adoption statuses (see Supplementary Table S7 for detailed regression results, and Supplementary Table S8 for post-estimation diagnoses). The model correctly identified 66% of adopters and 75% of non-adopters. However, to correctly identify the adopters is more important from a customer acquisition perspective, and logistic regression would likely miss more than 30% of the adopters.

**Figure 2:**
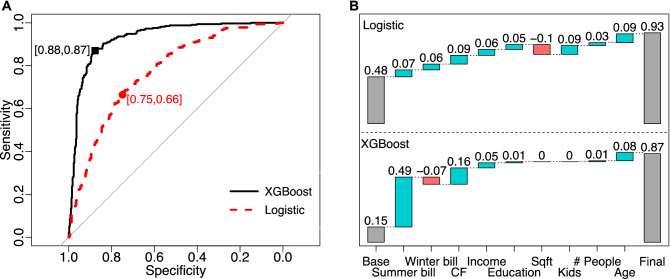
Comparisons of prediction performance and marginal impacts of logistic regression and XGBoost. (**A**) Receiver operating characteristic (ROC) curve with specificity and sensitivity from the 20% out-of-sample testing results. (**B**) Marginal impacts of variables or variable contributions to the predicted PV adoption probability for a representative household by increasing the mean household attribute levels (Base) by one standard deviation (Final); for categorical variables, the median and next level were used to calculate the change. CF, capacity factor. Sqft, square footage.

We further employed a popular machine learning algorithm—XGBoost (eXtreme Gradient Boosting), to predict PV adopters with the same set of household features as the logistic regression has. XGBoost uses an ensemble of decision trees to make a prediction and penalize model complexities at the same time (see Methodology for more details). Since the correlations among household features are not very high (Supplementary Table S5), we did not use any feature reduction or extraction method here (with another benefit of facilitating interpretation of later fitting results). The results indicate that machine learning surpasses the logistic regression in predictive performance. The predictive model correctly predicted 87% of the two PV adoption statuses, compared to 71% for logistic regression. The correct adopter rate increased from 66 to 87% and the correct non-adopter rate increased from 75 to 88% (Fig. 2A).

Figure 2B further compares the marginal impact of household attributes between logistic regression and XGBoost, which is defined as the relative contribution to changes in PV adoption probability resulting from increasing the variable mean by one standard deviation (SD). The logistic regression results in the top panel suggest that the representative household (i.e., average on all household-level attributes) is almost indifferent to solar PV adoption, with a predicted adoption probability of 0.48. The marginal effects of most household attributes are mostly 0.05–0.09. Increasing the summer and winter monthly bills by one SD increases the likelihood of becoming a PV adopter by 0.07 and 0.06, respectively. In XGBoost, by contrast, the same representative household is very unlikely to be a PV adopter (adoption probability only at 0.15). Increasing the summer bill by one SD increases adoption probability by 0.49, immediately making this household a PV adopter. However, increasing the winter bill by one SD lowers the adoption probability by 0.07 (for this household). Other factors such as house square footage and having kids at home have negligible effects in XGBoost but are among the most impactful factors in logistic regression. Such large differences between XGBoost and logistic regression point to important factors that determine the prediction performance of these two methods.

We further check the robustness of the enhanced predictive performance of XGBoost in several ways. First, we vary the variable set to test our choice of household attributes in modeling (Supplementary Table S7). Second, we re-run the same models using a smaller sample without any missing data imputed (Supplementary Fig. S3). Third, we reserve either 10% or 30% of the total sample as the testing dataset (Supplementary Table S9). Fourth, we vary the share of PV adopters in the dataset from 10 to 50% before any modeling (Supplementary Fig. S4). Fifth, we use one of the four states as the test set and the other three states as the training set to check the sample comparability across states (Supplementary Table S10). Lastly, we conduct a model uncertainty analysis with 30 different random seeds to split the raw dataset (Supplementary Fig. S5). The results of these robustness checks support the superior predictive performance of XGBoost over logistic regression. We also run other common classification and machine learning methods including linear and quadratic discriminant analysis, support vector machine, and random forest (Supplementary Table S11), and find that the better predictive performance of XGBoost remains apparent.

### Explaining the enhanced performance of XGBoost

**Figure 3:**
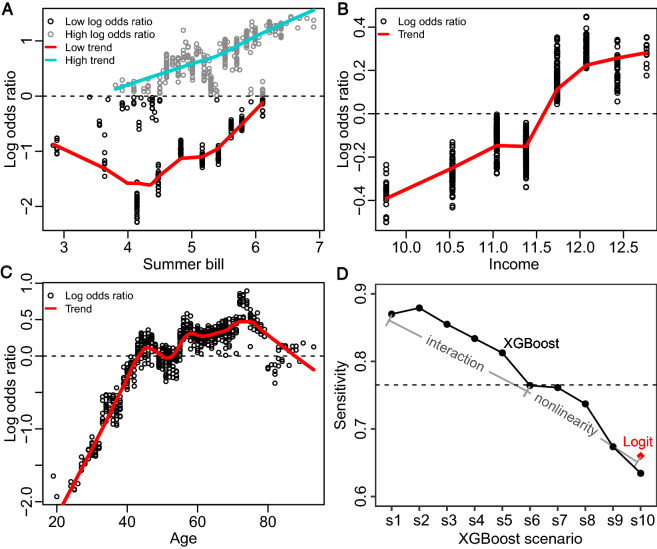
Explaining the enhanced performance of XGBoost over logistic regression: nonlinearity and variable interaction. (**A**) Nonlinear effect for summer monthly bill. (**B**) Nonlinear effect for household income. (**C**) Nonlinear effect for respondent’s age. (**D**) Decomposing the enhanced performance of XGBoost into variable interaction and nonlinearity. In (**A**–**C**), each circle represents one household in XGBoost, and localized trends are shown in colored curves; two different trends are shown for monthly summer bill that separate the log odds ratio by zero; the estimated effect by logistic regression would be a straight line in the figure; summer bill, and household income were log transformed. In (**D**), results in sensitivity for ten different scenarios (s1–s10) are shown for XGBoost (black solid circles), with the baseline logistic regression result in comparison (logit, red diamond).

We find that incorporating complex nonlinearity and variable interaction are the key reasons to explain the enhanced performance of XGBoost compared to logistic regression. While all household attributes have a linear marginal impact on the log odds ratio in the logistic regression, their impacts are much more complex in machine learning, as fundamentally determined by the decision tree structure in XGBoost (see Supplementary Fig. S1 for exemplary decision trees). Figure 3A–C shows the marginal effect for three continuous variables on PV adoption: summer bill, household income, and homeowner’s age. First, for households with a higher probability of adoption (log odds ratio > 0), a higher summer bill increases the adoption probability (Fig. 3a); however, for households with a lower adoption probability (log odds ratio < 0), the impact of electricity bills exhibits a U-shape pattern, suggesting that other household attributes are interacting with electricity bills (Supplementary Fig. S2). Second, though higher income levels are generally associated with greater odds ratio of adoption, the overall trend is curvilinear. Third, the three stages of age effects on PV adoption (i.e., 20–45, 45–75, and 75–90) are highly nonlinear, too.

In Fig. 3D, we further decompose the enhanced performance of XGBoost over logistic regression into two factors: variable interaction and nonlinearity, where we differentiated interactions between variables from nonlinearity within one variable. To separate the impact of these two factors, we designed 10 scenarios to run XGBoost and each scenario consisting of one specific parameter combination that represents a certain degree of variable interaction and nonlinearity (see Methods for detail, and Supplementary Table S12 for scenario definitions). After removing interaction and nonlinearity and simplifying the decision trees gradually, the predictive performance of XGBoost becomes similar to or even lower than that of logistic regression. The above finding indicates that variable interaction and nonlinearity are the two key reasons to explain the performance gap between these two methods. Specifically, removing variable interaction decreases XGBoost’s performance in sensitivity (i.e., true positive rate) from 87 to 76%, and removing nonlinearity further lowers its performance from 76 to 66%, the latter being the sensitivity of logistic regression. We focus on sensitivity mainly because it is the metric where XGBoost and logistic regression differ the most.

**Figure 4:**
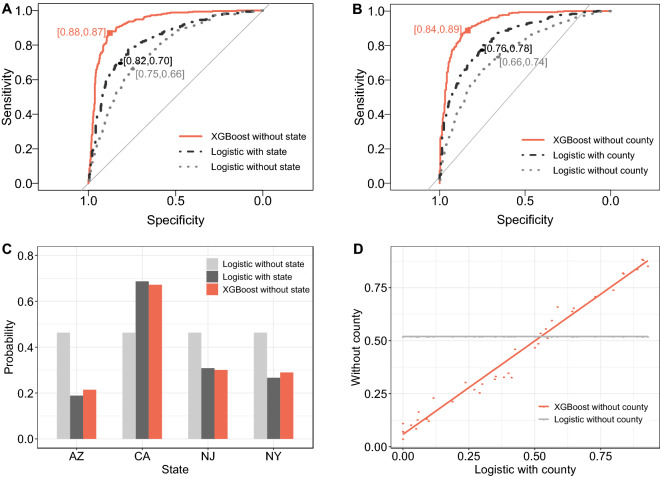
Key latent information captured by XGBoost: State and county of respondent. (**A**) Predictive performance with and without state information of respondents. (**B**) Predictive performance with and without county information of respondents. (**C**) State effect with and without state information of respondents. (**D**) County effect with and without county information of respondents. In county analyses, we only included counties with a sample size of at least 20 to make later train-validation-test data splits feasible, and only 40 counties (N = 2977) were included in (**B**,**D**). As a result, the predictive performance by XGBoost in (**D**) is different from that in (**C**). AZ, Arizona; CA, California; NJ, New Jersey; NY, New York.

Another reason to explain the improved performance of XGBoost is that it can potentially recover key latent information embedded in the data. For example, including geographical information such as the state or county of the respondent increases the prediction accuracy of logistic regression to some extent (Fig. 4A,B), but XGBoost with this additional information does not see a similar improvement (though still much better than logistic; see Supplementary Table S11). This is probably because the nonlinearity and interactions among household attributes in XGBoost already capture such information associated with regionality. As shown in Fig. 4C,D, even without including the state or county dummy variables in the model, XGBoost produces regional differences similar to or better than that from logistic regression explicitly with those dummy variables.

### Customer acquisition cost savings and new market opportunities

**Figure 5:**
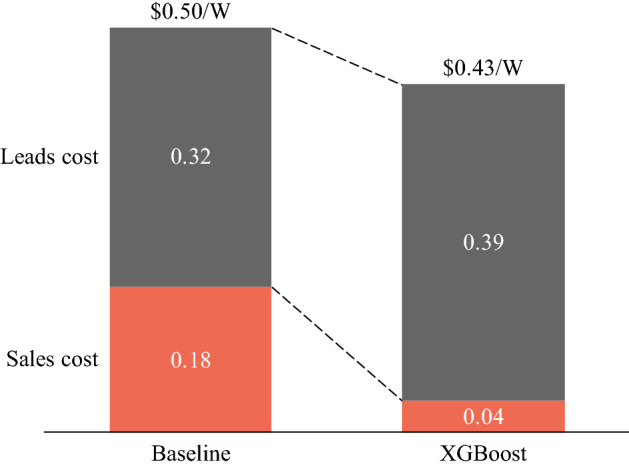
XGBoost saves customer acquisition costs that consist of leads cost and sales cost. The baseline case is where the installer company does not use any prediction results to selectively contact and visit potential customers. The result for logistic regression is omitted here since the installer company would rather not use prediction results from logistic regression (see Supplementary Note for details). The higher leads cost for XGBoost is to ensure it produces the same number of PV adopters in the end as the baseline case. Also note that the numbers shown in the figure mainly reflect the customer acquisition cost situation in California, which might differ for other states.

Leveraging the enhanced predictive performance of XGBoost, PV companies can better allocate their marketing resources, increase sales closing rates, and reduce customer acquisition costs. Rather than reaching out to all potential customers with different probabilities of PV adoption, the installer company can first tag customers into different groups based on our predictions, and then send more people to contact and visit the predicted PV adopters and fewer people to those predicted non-adopters. With good prediction results, sales cost can be saved in this way due to the enhanced closing rate, though at a price of consuming a larger pool of customer leads. Upon building a succinct cost saving model (see Methods and Supplementary Note for calculation details), we find that XGBoost can help PV companies reduce customer acquisition costs by about $390 per PV installation. Assuming an average system size of 5.6 kW, the cost saving in customer acquisition is roughly $0.07/Watt, which is around 15% of the total customer acquisition costs (Fig. 5). Such cost savings, if passed through to PV customers, would make rooftop PV more affordable to more people, expand the market size, and accelerate PV diffusion.

**Figure 6:**
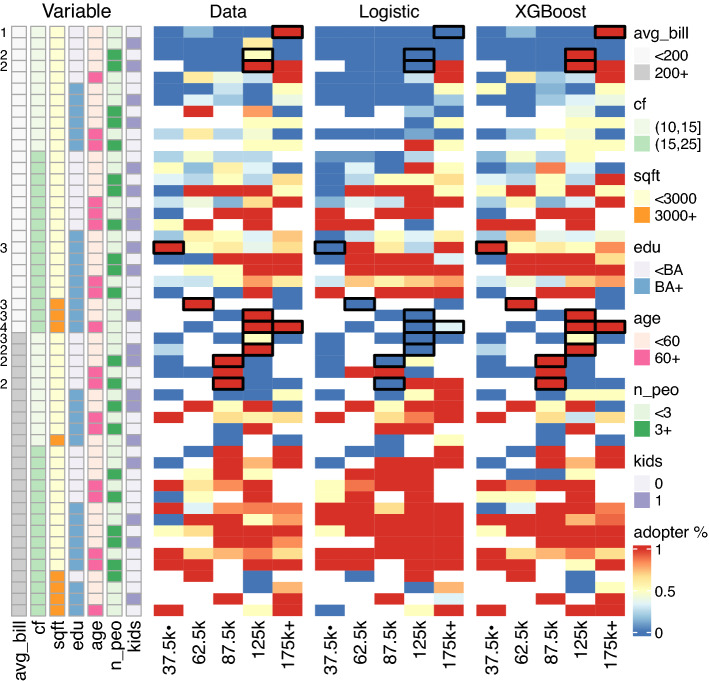
XGBoost identifies new market opportunities to PV companies relative to logistic regression. New market opportunities or segments are highlighted in black boxes, and they are correctly predicted by XGBoost (verified using preserved data), but somehow missed out by logistic regression. Each market segment is defined by a unique combination of household attributes. To make figure readable, we collapsed most household attributes (in plotting) into two levels except for household income (five levels). Numbers (1–4) shown on the very left are possible groups of these new market opportunities. Avg_bill, average of summer and winter electricity bills; cf, capacity factor; sqft, square footage; edu, education levels; age, homeowner’s age; n_peo, number of house occupants; kids, having kids at home or not.

New market opportunities could also emerge from machine learning. By comparing the predictive performance of XGBoost and logistic regression for each market segment (i.e., a unique combination of household attributes), we find that XGBoost can identify new market opportunities by predicting PV adopters in those segments correctly, which however would be missed out by logistic regression. Figure 6 highlights potential new market opportunities identified by XGBoost in black boxes, and we further classify them into four groups by examining the dominant factor behind XGBoost’s successful prediction of PV adoption. Groups 1 and 2 are those households mostly driven by income and electricity bills, respectively, group 3 is mostly driven by solar irradiation resources (i.e. ‘cf’), and the age variable played a vital role in increasing the odds for group 4. These determining factors are similar to what we have seen in Fig. 2B, in which the marginal impact from a single variable (e.g., summer bill) can sometimes make an overturning difference in adoption probability. By contrast, the quasi-linear effects in logistic regression imply that simultaneous and mostly equal contributions from many household attributes are required to overcome the non-adoption inertia, making its (modelled) adoption process more difficult. In short, PV companies could use insights from machine learning to identify market opportunities that were previously overlooked.

## Discussion

Technology adoption decisions are notoriously difficult to understand and predict, in part because decisions depend on interacting technical, economic, social, and individual factors^13,34^. In a similar vein, soft costs and customer acquisition costs are notoriously difficult to reduce due to the challenges in predicting forthcoming adopters. Our study leveraged machine learning algorithms that can capture the nonlinearity and complicated interactions among household attributes to improve the prediction performance of PV adoption statuses. An enhanced prediction can help reduce PV soft costs and accelerate adoption and its associated societal benefits. One key feature of our study is that we only relied on the most common socio-economic indicators in the literature^13^, such as income, education and age, along with monthly electricity bill information. The advantage of using these variables is that they are highly accessible so that PV companies can collect data on them with little cost. Consequently, our methods and results can be easily applied by the solar PV industry.

Our major conclusion is that highly significant factors in explanatory models are not necessarily good predictors for PV adoption, echoing recent advances in statistics and statistical learning^35,36^. Similarly, highly significant regression models (e.g. based on *F* statistics) are not necessarily good predictive models, largely because of researchers’ focus on the marginal effect of certain variables and the simplified way these variables are introduced into the model. Although more and more variables are found to be significantly associated with PV adoption, the marginal contribution of each additional variable to our understanding of PV adoption is somewhat diminished. Furthermore, the threat of over-fitting cannot be underestimated with a relatively small sample size, especially without out-of-sample testing. Our findings suggest that there is a strong need to shift from significance-based to prediction-oriented methodologies, not only for PV adoption studies, but also for studies on other clean technologies, for which adoption behavior is crucial to realizing their full social and environmental potential.

One implication of our study is that data science and machine learning can be used to reduce solar PV soft costs and broaden the market size^7,8^. By successfully identifying potential PV adopters, machine learning techniques can substantially reduce customer acquisition costs and identify new market opportunities for PV companies. According to our estimation, using XGBoost to identify potential customers can reduce customer acquisition costs by about 15% or 7¢/Watt. These cost savings, if passed on to PV customers, would make rooftop PV more attractive to more people. For installers operating under a fixed marketing and sales head count, these cost savings would also enable the company to reach a greater number of potential adopters, which can be then facilitated by the new market opportunities identified by machine learning algorithms. This is especially important given the increasing number of markets in which PV companies encounter more saturation among existing customers^53^.

Concerns may arise about whether using the prediction results on PV adopters would perpetuate a preference for like customers rather than diversifying those customers (the so-called “information cocoon” problem^54^). As a response, PV companies could combine machine learning with other customer acquisition strategies to diversity their customer base. First, rather than discarding those predicted non-adopters all, companies can work with social media platforms or advertising firms to target and retrieve promising non-adopters, similar to what other companies would do for their recommender systems^55,56^. In examining the adoption probability for predicted non-adopters from XGBoost, we do find that actual (but missed) PV adopters had relatively high adoption probability compared to actual non-adopters, with a mode at around 0.42 (Supplementary Fig. S6). Second, companies can organize community events in the local area of predicted adopters, and offer strong incentives for them to refer their friends, once they have become actual PV adopters. By retrieving promising predicted non-adopters or leveraging correctly predicted adopters’ social network, companies are able to move beyond specific machine learning algorithms and have a more diversified customer base.

Another related concern is that machine learning algorithms may reinforce the current inequalities in solar adoption^53,57,58^. This risk could be mitigated in two major ways. First, applying predictive models can reduce PV prices, making PV more accessible to underserved markets. Second, policymakers and PV companies could use predictive models to identify households in the disadvantaged groups with a high probability of adoption. According to our results, we find that machine learning algorithms are able to predict PV adopters more accurately across all socio-economic statuses (Supplementary Fig. S7). By targeting specific households, policymakers or installers could “seed” adoption in disadvantaged communities^53^ (Supplementary Table S13 further demonstrates that XGBoost improves the accuracy of predicting low-income adopters).

Broadly speaking, improving the predictive accuracy of adoption models could also help electric system planners with forecasting where and how much future PV adoption will occur. It could also be useful for regulators and policymakers who develop policies and incentive structures for distributed PV customers, as well as when estimating their costs and potential environmental impacts. For example, access to a predictive model combined with peer effects that spur more solar adoption^59,60^ could increase the efficacy of subsidies that target specific geographic locations, including low-income communities^53,58,61^. Furthermore, data mining and machine learning methods can be utilized to reduce soft costs for contract cancellation, supply chain management, labor assignment, and permitting and inspection issues. Machine learning algorithms can also be employed to improve adoption of other distributed energy technologies, such as heat pumps, electric vehicles, and battery storage.

Several limitations are worth noting. First, admittedly, our data are not random samples, and the four studied states may not be fully representative of the entire United States. This may reduce the generalizability of our prediction results to other states. However, based on the results of multiple robustness checks, we believe that the machine learning algorithms’ better performance is resilient to the sampling strategy and a larger geography compared to the significance-based methods. Second, since we only have cross-sectional data, we could not fully test whether the machine learning algorithm could retain its predictive power over time. However, we observed that the predictive power for PV adopters increased over the course of our sample period from 2011 to 2015 (Supplementary Fig. S8). Third, this study leverages the most visible and objective economic factors in our prediction; future studies could include other subjective variables (e.g., green premiums that consumers are willing to pay for renewable energy^62^, and responsible electricity consumption behaviors^63^) in the model to achieve an even better predictive performance (albeit with higher data costs). Lastly, after identifying potential PV customers, companies could even try different nudging tools (monetary and non-monetary tools such as a subsidy for self-consumption and information campaigns on the economic and environmental advantages of green technologies^64^) to better incentivize them in the transition of solar PV installation.

## Data and methods

### Data

Our survey data of PV adopters and non-adopters at the household level were collected by the National Renewable Energy Laboratory (NREL) between June 2014 and April 2015 as part of its Solar Energy Evolution and Diffusion Studies (SEEDS) project. The Institutional Review Board (IRB) at Portland State University, a key collaborator with the NREL SEEDS project, provided approval to collect our data. All research was performed in accordance with relevant guidelines and regulations, and informed consent was obtained from all survey participants. The data sample comprised single-family United States households in California, Arizona, New Jersey, and New York. We chose these four states for two reasons: (1) they were the top four residential PV markets in 2014 in the U.S.^65^, and (2) national installers played a big role in these states. In each state, survey participants were primarily voluntary respondents identified by installers and lead-generator companies that collaborated on the research project, supplemented by paid respondents (i.e., panelists recruited through a web-panel company). A minimum of 100 responses per state per customer type were collected. Multi-family households and renter-occupied houses were excluded because of their low probability of adopting solar PV.

Survey items asked about respondents’ monthly winter and summer electricity bills, various socio-demographics (e.g., income, education, age, household size, etc.), motivations for considering PV, and experiences surrounding their decision of PV installation. We further complemented the survey data with zip-code level solar irradiations in PV capacity factor that we sourced from NREL’s PVWatts. Although there were both objective and subjective survey items, this study focuses on a subset of the objective items to build our predictive models, i.e., nine highly visible and easy-to-measure household attributes that can be easily obtained by PV companies. The purpose of doing so is to build a parsimonious empirical model with high out-of-sample prediction performance and low data collection costs.

There were 3,592 responses in our dataset, which represents the largest sample in the literature on PV adopters and non-adopters at the household level (Supplementary Table S1). 48%, 19%, 17%, and 16% of the respondents are from California, New York, New Jersey, and Arizona, respectively. There are about 30% of the data with missing value for key variables that were needed for our data analysis. The missing data rate was roughly the same across the four states: 30% for California, 29% for New Jersey, 32% for New York, and 23% for Arizona. The relatively low missing data rate for Arizona resulted from using more paid respondents to guarantee the required sample size by sub-category. After deleting 22 cases due to invalid or missing regional information, we used multiple imputation to obtain 3,570 responses for the final data analysis; 46% of them are adopters and 54% are non-adopters (see Supplementary Table S2 for sample breakdown by state). The summary statistics for the data are shown in Supplementary Table S3.

### Methods

The overall methodology is summarized in the flow chart in Supplementary Fig. S9. To better predict which households were likely to adopt solar PV, we first split the total sample into training, validation, and test datasets. In all model runs, we pre-reserve 20% of the data for out-of-sample testing purposes. For machine learning algorithms, five-fold cross-validation for hyperparameter tuning was also used to ensure that the validation dataset has a similar sample size to the testing dataset.

We choose logistic regression and XGBoost as our two baseline prediction methods, since the former is the typical significance-based method used in the literature and the latter is an award-winning machine learning algorithm with great prediction performances. We have used three criteria for feature selection: (1) household attributes must be objective, highly visible and easy-to-measure, so that data collection costs for PV companies are low; (2) household attributes must be commonly seen in the PV adoption literature; and (3) the selection of household attributes cannot bias toward neither of the two methods being compared. As to feature transformation, we took the logarithm of electricity bills and household income to reduce their distributional skewness (Supplementary Table S4) and to obtain better predictive performances (mainly for logistic regression); applying the Box-Cox transformation to remove skewness produces very similar prediction results to what we obtain with the log transformation (Supplementary Table S4). Categorical variables such as education levels and house size are dummy coded beforehand.

#### Prediction rate

Other than the above two classification methods we use to model households’ PV adoption statuses, below we also employ the method developed by Lo et al.^35^ to calculate the prediction rate of a single household attribute to determine whether it was a strong predictor of the binary outcome variable. For example, given the binary variable statuses $A$ and $N$, where $A$ is adopter and $N$ is non-adopter, the observed value of the single household attribute $x$ comes from the distribution either ${f}_{A}$ or ${f}_{N}$. Assuming the costs of false positives and false negatives are equal, an appropriate Bayes rule to decide the outcome status for $x$ is in favor of the larger value of ${f}_{A}(x)$ and ${f}_{N}(x)$. When $x$ is discrete and takes a finite number of values, the corresponding error rates for the above Bayes rule are ${\sum }_{x:{f}_{A}\left(x\right)<{f}_{N}(x)}{f}_{A}(x)$ and ${\sum }_{x:{f}_{N}\left(x\right)<{f}_{A}(x)}{f}_{N}(x)$, respectively. The average error rate is then 0.5*${\sum }_{x}min\left({f}_{A}(x) ,{f}_{N}(x)\right)$. As a result, the prediction rate is 1 minus the average error rate:

1 $$\mathrm{Prediction rate}=0.5*{\sum }_{x}max\left({f}_{A}(x) ,{f}_{N}(x)\right)$$

For continuous variables, Eq. (1) can be rewritten with integrals instead of summations.

#### Logistic regression

Logistic regression (or logit regression) is a statistical model that uses a logistic function to model binary dependent variables. In our case, such a binary dependent variable is whether or not a household had adopted solar PV. We chose logistic regression as a comparison model to the machine learning algorithms because logistic regression is the most commonly used method in the previous PV adoption research.

Denote the probability of a PV adopter as $p$, and the original dependent variable as $Y$, then we have $p=Prob\left(Y=1\right)$. Logistic regression further uses the log odds of the event when $Y=1$ as the new dependent variable, and assumes a linear relationship with a vector of household attributes ${X}_{i}$ on the right side of the regression model:

2 $$\mathrm{log}\left(\frac{{p}_{i}}{1-{p}_{i}}\right)=\alpha +{\beta }^{^{\prime}}{X}_{i}+{\varepsilon }_{i}$$

where $\alpha$ is the intercept, $\beta$ is the coefficient vector, and ${\varepsilon }_{i}$ is the error term. Since $0\le p\le 1$, the logistic function of $\mathrm{log}\left(\frac{{p}_{i}}{1-{p}_{i}}\right)$ thus converts $p$ to a real value in the range of $\left[-\infty ,+\infty \right]$. The goal of logistic regression is to identify which of $X$ has a significant impact on $Y=1$ (i.e., becoming a PV adopter in this case), while keeping all other factors constant. The magnitude of these marginal impacts is captured by the coefficient vector $\beta$. However, the assumed linear relationship between $X$ and the log odds of the event may be constraining with respect to the ultimate predictive performance.

#### XGBoost

XGBoost (extreme gradient boosting), is a typical gradient tree boosting method widely used in machine learning challenges such as the Kaggle competition, which outperforms other classic methods such as naïve Bayes, random forest, and support vector machine. More than half of the challenge winning solutions used XGBoost, which is even more popular than deep neural nets. The following description of this method is mainly drawn from^66^, with certain re-organizations.

One of the defining features of XGBoost is to use an ensemble of decision trees to boost performance. Assuming in the end that there are $K$ trees in the ensemble, each with a predicting score ${f}_{k}$ for subject $i$ with features ${X}_{i}$, then the overall predicting score for this subject is the sum of all $K$ predicting scores: ${\widehat{y}}_{i}={\sum }_{k=1}^{K}{f}_{k}\left({X}_{i}\right)$.

The goodness of fit for an ensemble of decision trees is defined as:

3 $$\mathcal{L}={\sum }_{i=1}^{n}l\left({y}_{i},{\widehat{y}}_{i}\right)+{\sum }_{k=1}^{K}\Omega \left({f}_{k}\right)$$

where $l$ is the training loss between the observed outcome ${y}_{i}$ and its predicted version ${\widehat{y}}_{i}$, and $\Omega$ is the regularization term that captures the complexities of all $K$ trees. By default, a quadratic loss function and L2 norm are used in XGBoost, which we used in this study. Specifically, $l\left({y}_{i},{\widehat{y}}_{i}\right)={\left({y}_{i}-{\widehat{y}}_{i}\right)}^{2}$ and $\Omega =\upgamma T+\frac{1}{2}\lambda {\sum }_{j=1}^{T}{\omega }_{j}^{2}$, where $T$ is the number of leaves, ${\omega }_{j}$ are leaf scores, and $\upgamma$ and $\lambda$ are parameters to be calibrated. As such, $\Omega$ penalizes the complexity of the tree model. Furthermore, prediction function ${f}_{k}$ and leaf scores are related in that: ${f}_{k}\left({X}_{i}\right)={\omega }_{q({X}_{i})}$, where $q$ is the tree structure that maps subject $i$ to one of the $T$ leaves. All subjects in the same leaf have the same score.

The predictive performance of a tree proceeds in two major steps: first it finds the best prediction function and leaf scores given the tree structure; second it finds the best tree structure. For a for a fixed tree structure $q\left({X}_{i}\right)$, one can compute the optimal weights or leaf scores ${\omega }_{j}^{*}=-\frac{\sum_{i\in {I}_{j}}{g}_{i}}{\sum_{i\in {I}_{j}}{h}_{i}+\lambda }$, and then the optimal objective value becomes:

4 $${\widetilde{\mathcal{L}}}^{\left(t\right)}\left(q\right)=-\frac{1}{2}\sum_{j=1}^{T}\frac{{\left(\sum_{i\in {I}_{j}}{g}_{i}\right)}^{2}}{\sum_{i\in {I}_{j}}{h}_{i}+\lambda }+\upgamma T$$

where ${g}_{i}$ and ${h}_{i}$ are the first and second order gradient statistics on the loss function $l$. Equation (3) is similar to the impurity score for a decision tree.

Next the best tree structure is identified $q\left({X}_{i}\right)$. XGBoost uses a greedy method to grow the tree. It starts with a tree with one node and zero depth. Then, for each node of the tree, it enumerates the overall features; for each feature, it sorts by feature values and then decides the best split along that feature; and takes the best split across all features. The best split will maximize the increase in the objective value of the tree. For example, if one splits a sorted ${x}_{i}$ into a left half ($L$) and a right half ($R$), then the increase in the objective is:

5 $$\Delta \widetilde{\mathcal{L}}=\frac{1}{2}\left[\frac{{G}_{L}^{2}}{{H}_{L}+\lambda }+\frac{{G}_{R}^{2}}{{H}_{R}+\lambda }-\frac{{\left({G}_{L}+{G}_{R}\right)}^{2}}{{H}_{L}+{H}_{R}+\lambda }\right]-\upgamma$$

where ${G}_{L}=\sum_{i\in L}{g}_{i}$ and ${G}_{R}=\sum_{i\in R}{g}_{i}$, and similarly for ${H}_{L}$ and ${H}_{R}$. Obviously, this greedy method to grow trees can be computationally expensive; however, XGBoost uses an approximate algorithm, caching-aware prefetching algorithm, and parallel learning to enhance speed.

To achieve strong out-of-sample prediction performance without penalizing the in-sample model fitting performance, we used Bayesian optimization (*R* package ‘ParBayesianOptimization’ v1.2.4^67^ in its original code) to tune hyperparameters in XGBoost, namely the maximum depth of a tree, eta (learning rate), gamma (minimum loss reduction to make a further partition), minimum child weight (minimum sum of weight in a leaf node), column sampling ratio (ratio of columns when constructing each tree), and row sampling ratio (ratio of rows when constructing each tree), as well as the number of rounds for boosting. We then used the best set of hyperparameters to re-train the XGBoost model and predict out-of-sample PV adoption outcomes. We also use an *R* package ‘xgboostExplainer’ v0.1^68^ to probe into XGBoost results and make them more interpretable by calculating the impact of each feature on the prediction at the leaf level. We further make waterfall graphs out of these marginal impacts and examine the nonlinearity of these impacts.

*Decomposing XGBoost*. In order to decompose the enhanced prediction performance of XGBoost over logistic regression, we carefully designed 10 scenarios (Supplementary Table S12) to re-run XGBoost. The goal is to control for variable interaction first, and then for nonlinearity, since these are the two major factors that determine the better performance of XGBoost.

The definition of specific scenario and parameter setting are as follows: First, we restrained the degree of variable interactions used in the decision trees of XGBoost via two parameters called “colsample” (column sampling ratio) and “round” (number of rounds for boosting). For example, using 0.07 instead of 1 for “colsample” means that every decision tree will randomly draw at maximum 7% of all columns (i.e., household attributes) from the dataset; since there are 15 columns in our baseline model, it means only one variable will be used in growing each decision tree. This will ensure that no interaction is allowed within one decision tree. However, because XGBoost uses an ensemble of decision trees to fit the model and make a prediction, adding up prediction scores from many decision trees implies variable interaction across trees. That is why we also need to restrain the number of decision trees used in XGBoost via the parameter ‘round’ to further remove variable interactions. By limiting the maximize number of the allowed decisions trees from 1000 to 20 (almost one decision tree for one variable; results from using 15 are very similar), we can make sure that only very limited variable interaction remains in XGBoost. We cannot remove all the interactions by limiting the number of decisions trees to be one since in that case with colsample equal to 0.07, we only used one variable to fit the whole model.

Second, we restrained the degree of nonlinearity through the parameter “depth” (maximum depth of a tree) in XGBoost. Since every additional tree depth means that we add more branches to the tree as a new layer, it translates directly to more nonlinearity even with a single variable being used to grow the tree. Meanwhile, with more variables in use, higher tree depth would also leverage those other variables to growth the tree, resulting in interactions among variables. That is why we first need to restrain “colsample + round” and then “depth” in the process, and not vice versa. After limiting the tree depth to be four, the prediction performance of XGBoost becomes very similar to that of logistic regression.

Thus, by restraining the above three parameters in XGBoost, we are able to control the degree of nonlinearity and interactions included in this method, peeling off its complexity step by step, and in the end making XGBoost similar to classic methods like logistic regression.

#### Cost savings in customer acquisition

The current high customer acquisition costs are the results of two major components: (1) leads cost, and (2) sales cost. The leads cost is for an installer company to purchase solar leads from a lead generation company, and the sales cost is to pay the sales staff for contacting and visiting potential customers and their commissions^69^. After receiving the leads information from the lead generation company, the installer company will contact all those leads and makes an appointment with some of the leads. Only a small proportion (e.g. 7%) of leads will become final sales (see Supplementary Note for detailed numbers). Machine learning predictions can be used by the installer company to enhance its sales closing rate (or the leads conversion rate). Based the prediction results, installers are able to tag and group potential customers into predicted PV adopters and non-adopters, and then send more people to contact and visit all the predicted PV adopters and fewer people to those predicted non-adopters.

The calculation of cost savings in customer acquisition are based on the following three rules: (1) its sales staff should contact and visit (visit for short thereafter) all tagged PV adopters, while only visiting some tagged non-adopters in proportion to the adopter share in this group:

6 $$\begin{aligned} & Visit_{A} = Tag_{A} \\ & Visit_{N} = { }Visit_{A} \times \frac{{Share_{N} }}{{Share_{A} }} \\ \end{aligned}$$

where ${Visit}_{A}$ is the number of household visits to tagged adopters—${Tag}_{A}$, ${Visit}_{N}$ is the number of household visits to tagged non-adopters, ${Share}_{N}$ is the adopter share in the tagged non-adopter group, and ${Share}_{A}$ is the adopter share in the tagged adopter group.

The second rule is that: (2) if the final payoff from visiting the tagged non-adopter group is much less than one sale or installation (say 0.3 or 0.4 in the statistic sense), the installer company would rather not visit any tagged non-adopters.

7 $${Visit}_{N}^{^{\prime}}=0 if {Visit}_{N}*{Share}_{N}<0.5$$

The third rule is that: (3) the final payoff or number of sales should be the same for different methods in comparison:

8 $${Visit}_{A}*{Share}_{A}+{Visit}_{N}^{^{\prime}}*{Share}_{N}=B$$

where $B$ is the required sales to be fulfilled whether the installer company uses tagging and selective visiting or not.

The above rules assume that the installer company is risk-neutral and rational, and its allocation of human resource is based on the expected payoffs. With these rules, the total cost of household visits is: ${c}_{1}*({Visit}_{A}+{Visit}_{N}^{^{\prime}})$, where ${c}_{1}$ is the unit visiting cost. The total leads cost is: ${c}_{2}*\left({Tag}_{A}+{Tag}_{N}\right)$, where ${c}_{2}$ is the unit leads cost, and ${Tag}_{N}=\frac{{Visit}_{N}}{{Share}_{N}}$. These two costs add up to the total customer acquisition costs (see Supplementary Note for calculation details).

## Supplementary Information

Supplementary Information.

## Data Availability

The original survey and cleaned dataset used for the current study are available in the github repository, (https://github.com/rosenbloog/PV_Machine_Learning). A sample (*N* = 30) of the dataset is presented in Supplementary Table S14. Codes used in the analyses of the datasets are available from the corresponding author on reasonable request.
