# Supplementary material for: Machine learning reduces soft costs for residential solar photovoltaics
Source: Sci Rep. 2023 May 3;13:7213. doi: 10.1038/s41598-023-33014-4 (PMC10156750; doi:10.1038/s41598-023-33014-4)
Supplement: Supplementary file 1 — Supplementary Information. [file 41598_2023_33014_MOESM1_ESM.docx]

**Supplementary Information for**

**Machine learning reduces soft costs for residential solar photovoltaics**

**Changgui Dong, Gregory Nemet, Xue Gao, Galen Barbose, Benjamin Sigrin, Eric O'Shaughnessy**

**Changgui Dong**

**E-mail: changgui.dong@ruc.edu.cn**

**This PDF file includes:**

Supplementary note

Figs. S1 to S10

Tables S1 to S13

SI References

**Supplementary Note**

**A cost saving model for machine learning algorithms to reduce customer acquisition costs**

Customer acquisition costs mainly consist of two components: 1) leads cost, and 2) sales cost. The leads cost is for an installer company to purchase solar leads from a lead generation company, which is the typical industrial practice nowadays, whereas the sales cost is to pay the sales staff for contacting and visiting potential customers and their commissions.

According to SolarReviews (https://www.solarreviews.com/solar-leads/how-much-do-solar-leads-cost-in-each-state; last accessed: 2021-04-26), with a 7% leads conversion rate in California (used as an exemplary state), the installer company needs to purchase 14 solar leads from a valid source to generate one final sale and PV installation. After receiving the leads, the company first makes contact with all those leads, and then offers a quote to and makes an appointment with some of the leads, and finally one lead is converted to sales on average. The following figure summarizes the whole process, with conversion rates attached to each sub-process. In a word, although the installer company contact all 14 leads, only 10 of them would respond, and then four agree to set up an appointment with the company for a home visit; finally, only one PV installation materializes (https://www.solarreviews.com/solar-leads; last accessed: 2022-02-26).

The whole leads conversion process is multi-stage, iterative, and difficult to analyze, even more so without detailed cost data. For the purpose of this paper and to simplify things, we focus on the stages since leads become contacts (ignoring the first stage and thus a conservative estimate). Furthermore, we assume that the company’s salespersons take charge of the rest of the process: i.e., offering a quote, making an appointment, paying home visits and closing the sale. This is mostly true for small and middle installer companies. Even if salespersons are only responsible for home visits and onsite sales, to the extent that in-house staffs earn a similar wage to those salespersons, our assumption should not impact the final result. As a result of this assumption, the sales closing rate is about 10%, i.e., one sale out of 10 contacts.

On the cost front, it was estimated that one good lead costs $135 in California; with a 7% leads conversion rate, the cost of leads per sale is $1,929. Similarly, the leads cost per contact is $193 ($135/70%). Regarding sales cost, the average cost paid to salespersons is $0.18/Watt; with an average PV system size of 5.6 kW, the sales cost per system is $1,008 (per 10 contacts), or $101/contact.

The installer company can use machine learning prediction results of PV adoption to lower its customer acquisition costs by reducing futile contacts and home visits, and thus enhancing its sales closing rates. The current practice is that the company asks its salespersons to contact all potential customers without differentiating them first. This can be improved by using our prediction results to tag and classify them into predicted PV adopters and non-adopters, and these two groups will have different possibilities of adopting PV in the end. The installer company can then make better use of its salesforce by sending more people to contact and visit all the predicted PV adopters and fewer people to contact and visit those predicted non-adopters. With good prediction results, sales cost can be saved with enhanced sales closing rates. However, without any useful predictions at its disposal, the installer company has to contact and visit (visit for short thereafter) all potential customers. This is the baseline case we are going to discuss first.

**Baseline Case.** We use two matrices (i.e., the probability matrix and visit matrix) to show how to better allocate the installer company’s salesforce to tagged PV adopters and non-adopters. The first is a probability matrix (or a confusion matrix), which captures the installer company’s prior prediction, if any, of future PV adopters and non-adopters. For example, starting with a random guess, we have the following 2x2 probability matrix filled with 0.5. One reads the probability by columns (same for by rows) and the two probabilities in the same column add up to 1 (shaded row at the bottom); in other words, the two probabilities in a column reflect the relative prediction accuracy for each actual adoption group.

| Probability |  | Actual | |
| --- | --- | --- | --- |
|  |  | No | Yes |
| Predicted | No | 0.5 | 0.5 |
|  | Yes | 0.5 | 0.5 |
|  |  | 1 | 1 |

In the second and visit matrix below and together with the above probability matrix, the installer company will split the total household visits by its salespersons, say 100 times, among the predicted adopters and non-adopters. Assuming the actual composition of PV adopters and non-adopters is 1:9 (same hereafter), which is consistent with the 10% sales closing rate, the company would hit 10 real adopters in a pool of 100 contacts. Since the probability matrix is filled with 0.5, half of the actual PV adopters (second column in the probability matrix) will be tagged as predicted PV adopters, and the other half will be tagged as predicted non-adopters. Same for the actual PV non-adopter group.

What is more, the predicted adopter and non-adopter groups will each have the same actual adopter share, i.e. 10% (=5/50). Thus, the installer company will treat these two groups equally and split the 100 household visits evenly among them. The final payoff of such splitting is 5 adopters from each group, and 10 adopters in total. The success rate of household visits is 10%, i.e. the sales closing rate.

| Visit |  | Actual | | Calculation | | | |
| --- | --- | --- | --- | --- | --- | --- | --- |
|  |  | No | Yes | Tagged | Adopter share | # Visits | Payoff |
| Predicted | No | 45 | 5 | 50 | 10% | 50 | 5 |
|  | Yes | 45 | 5 | 50 | 10% | 50 | 5 |
|  |  | 90 | 10 | 100 |  | 100 | 10 |

Before we discuss the extent to which using good prediction results of PV adoption can save the installer company time and efforts, let us introduce the ideal case first.

**Ideal Case.** In this case, the installer company can predict the PV adopters and non-adopters perfectly well. As such, the probability matrix has 1 as its diagonal elements and 0 otherwise. In the visit matrix, the tagged PV adopters and non-adopters are 100% accurate; as a result, the installer company only needs to visit those 10 tagged PV adopters, with 10 PV adoptions as its payoff. The success rate of household visits is 100%; so is the sales closing rate.

| Probability |  | Actual | |
| --- | --- | --- | --- |
|  |  | No | Yes |
| Predicted | No | 1 | 0 |
|  | Yes | 0 | 1 |
|  |  | 1 | 1 |

| Visit |  | Actual | | Calculation | | | |
| --- | --- | --- | --- | --- | --- | --- | --- |
|  |  | No | Yes | Tagged | Adopter share | # Visits | Payoff |
| Predicted | No | 90 | 0 | 90 | 0% | 0 | 0 |
|  | Yes | 0 | 10 | 10 | 100% | 10 | 10 |
|  |  | 90 | 10 | 100 |  | 10 | 10 |

**Logistic Case.** Using the prediction results from the logistic regression in the paper, we obtain the probability matrix as follows. The true positive rate is 66% and the true negative rate is 75%, from which we can derive the false positive rate at 34% and the false negative rate at 25%.

| Probability |  | Actual | |
| --- | --- | --- | --- |
|  |  | No | Yes |
| Predicted | No | 0.75 | 0.34 |
|  | Yes | 0.25 | 0.66 |
|  |  | 1 | 1 |

As to the visit matrix, we need to set up several rules for the installer company first: 1) Its sales staff should visit all tagged PV adopters, while only visiting some tagged non-adopters in proportion to the adopter share in this group; 2) if the final payoff from visiting the tagged non-adopter group is (much) less than one sale or installation (say 0.3 or 0.4), the company would rather not visit any tagged non-adopters, and 3) the final payoff should be the same as the baseline case, i.e., 10 PV installations. Rule #1 can be better expressed using the formula below:

$${Visit}_{N}= {Visit}_{A}\times\frac{{Share}_{N}}{{Share}_{A}}$$

where ${Visit}_{N}$ is the number of household visits to tagged non-adopters, ${Visit}_{A}$ is the number of household visits to tagged adopters, ${Share}_{N}$ is the adopter share in the tagged non-adopter group, and ${Share}_{A}$ is the adopter share in the tagged adopter group. We further modify ${Visit}_{N}$ as follows (rule #2):

$${Visit}_{N}^{'}=0 if {Visit}_{N}\times{Share}_{N}<0.5$$

The above rules assume that the installer company is risk-neutral and fully rational in the sense that it will split its salesforce in proportion to the adopter share in each tagged group. This proportionality setup makes sure that using prediction results better than a random guess but worse than the ideal case will have their sales closing rate (and cost savings) falling in between these two extremes, too.

Back to the logistic regression case, in order to obtain 10 PV adoptions in the end (rule #3), the installer company needs to have 150 contacts at hand (deducted backwards from obtaining 10 adopters in the end with cost minimization). Based on the 9:1 composition of actual PV non-adopters and adopters, there will be 15 of them being actual adopters and 135 being actual non-adopters. Combining this with the probability matrix above, the company will tag 44 of the 150 contacts as predicted adopters and the rest 106 as predicted non-adopters. The adopter shares in these two groups are 5% and 23%, respectively. Then, following the visiting rules that we set up earlier (rules #1 & #2), the installer company should only visit those 44 tagged adopters with an adopter share of 23%. The installer company would rather not visit any tagged non-adopters in this case, since the payoff of doing so is low (rule #2; predicted payoff at 0.43). The final number of total household visits is 44 (per 150 contacts) instead of 100 (per 100 contacts) in the baseline case, but at a price of 50 more contacts. Such a price of having more contacts and purchasing more solar leads is to make sure that the installer company obtains 10 PV adoptions in the end (rule #3).

| Visit |  | Actual | | Calculation | | | |
| --- | --- | --- | --- | --- | --- | --- | --- |
|  |  | No | Yes | Tagged | Adopter share | # Visits | Payoff |
| Predicted | No | 101 | 5 | 106 | 5% | 0 | 0 |
|  | Yes | 34 | 10 | 44 | 23% | 44 | 10 |
|  |  | 135 | 15 | 150 |  | 44 | 10 |

**ML Case.** In the machine learning (ML) case, we obtain better prediction results than logistic regression. The true positive and true negative rates are now 87% and 88%, respectively, making the other two elements in the matrix 13% and 12%, respectively. We then use the same rules as logistic regression to calculate the necessary number of contacts and to split the potential household visits. In the end, 120 contacts are needed to generate 10 PV adoptions (rule #3). Since the true positive rate is very high, the installer company only tags 23 households as predicted PV adopters and the rest 97 as predicted non-adopters. After visiting all 23 tagged PV adopters (per 120 contacts), 10 adopters can be expected with an adopter share of 43%. Furthermore, the installer company does not have to contact and visit any tagged non-adopters in this case, since the payoff of doing so is extremely low (rule #2; predicted payoff at 0.02).

| Probability |  | Actual | |
| --- | --- | --- | --- |
|  |  | No | Yes |
| Predicted | No | 0.88 | 0.13 |
|  | Yes | 0.12 | 0.87 |
|  |  | 1 | 1 |

| Visit |  | Actual | | Calculation | | | |
| --- | --- | --- | --- | --- | --- | --- | --- |
|  |  | No | Yes | Tagged | Adopter share | # Visits | Payoff |
| Predicted | No | 95 | 2 | 97 | 2% | 0 | 0 |
|  | Yes | 13 | 10 | 23 | 43% | 23 | 10 |
|  |  | 108 | 12 | 120 |  | 23 | 10 |

**Case Comparison.** The above four cases have very different cost implications. To better compare them, we summarize their results in terms of leads cost, sales cost, and the total of these two. All the calculations are self-explanatory below in the table, assuming that the average leads cost per contact at $193, and the average cost per household visit at $101. In the end, the machine learning algorithm could save installer company $3,917 ($29,400 - $25,483) per 10 PV installations, or $392 per installation. With an average system size of 5.6 kW, the cost saving in customer acquisition is roughly $0.07/Watt, which is around 39% of the sales cost, or 13% of the total customer acquisition costs ($3,917/$29,400). In contrast, although the logistic regression could help reduce sales cost significantly, the increase in leads cost is more substantial, making logistic regression an unattractive option for the installer company. Of course, the installer company can always give up any tagging based on logistic regression, if doing so increases the leads cost more than it decreases the sales cost.

| Case | # Contacts | # Household visits | Leads cost | Sales cost | Total cost |
| --- | --- | --- | --- | --- | --- |
| Baseline | 100 | 100 | $19,300 | $10,100 | $29,400 |
| Ideal | 100 | 10 | $19,300 | $1,010 | $20,310 |
| Logistic | 150 | 44 | $28,950 | $4,444 | $33,394 |
| ML | 120 | 23 | $23,160 | $2,323 | $25,483 |

The above cost saving by machine learning has broad industrial implications. First, the sales cost saving of $0.07/Watt means that the installer company could further pass it through to PV customers, making rooftop PV affordable to more people. Second, the sales cost saving also means that the installer company could use its saved manpower to reach out to more people and households, speeding up the PV technology diffusion process and the resulting environmental benefits. Third, the more needed contacts and solar leads further bring positive spillover effects to the lead generation companies. Note that if the installer company can save some of its leads cost, it will mean equivalent business loss to lead generation companies; thus, we do not need to discuss leads cost saving here.

Lastly, as to the concern that using machine learning algorithms may drain the customer base more quickly than the baseline, we believe that as long as the machine learning algorithm does not focus on a narrow customer base but predict PV adoptions from all socio-economic statuses, using improved prediction results by machine learning algorithms would be helpful to find new customers; this is exactly what we find in our final prediction results. After all, a cost saving of $0.07/Watt could increase adoption considerably as these savings could make sales into unserved geographies profitable.

**Fig. S1.** Three exemplary decision trees with different complexities (*A*), (*B*), and (*C*). In the end, XGBoost uses an ensemble of several hundreds of simple decision trees to classify households into PV adopters and non-adopters.

**Fig. S2.** Interaction effect between electricity bills and other household attributes. Left panel shows that high winter bill (above the mean) is needed to increase the log odds ratio of summer bill above zero, and the right panel shows that high income (above the mean) is also needed to increase the log odds ratio of winter bill above zero.

**Fig. S3.** Comparisons of prediction performance and marginal impacts of logistic regression and XGBoost for complete samples without missing data imputed (N = 2,555). (*A*) Receiver operating characteristic (ROC) curve with specificity and sensitivity from the 20% out-of-sample testing results. (*B*) Marginal impacts or variable contributions to the predicted PV adoption probability for a representative household by increasing the mean household attribute levels (Base) by one standard deviation (Final); for categorical variables, the median and next level were used to calculate the change. CF, capacity factor. Sqft, square footage.

**Fig. S4.** Sensitivity analysis on the effect of different ratios of PV adopters to non-adopters on the baseline model results. We choose five data splits ranging from 10/90 to 50/50 to match the real-world leads conversion rate (~10%) or salespeople’s highest closing rate (~50%). To generate these data splits, we draw random samples from the fewer category and use all samples from the other category to match the required ratio.

**Fig. S5.** Model uncertainties in logistic regression and XGBoost. Each circle (blank or solid) represents one of the 30 model runs with different random seeds to split the data, whereas each line connects one pair of models in the same run.

**Fig. S6.** Predicted adoption probability for predicted non-adopters by XGBoost. Right panel further graphs the density function of adoption probability by adoption status.

**Fig. S7.** Comparison of predictive performance between logistic regression and XGBoost along socio-demographic variables. Value levels are compressed for summer electricity bill and household income to make the graph more compact.

**Fig. S8.** Predicted probability of adoption for PV adopters by actual adoption year. The increased probability of adoption over time for actual adopters suggests that the predictive performance of the two methods can be extended to the near future, especially considering that more people will adopt PV for economic rather than environmental reasons [29,63,64].

**
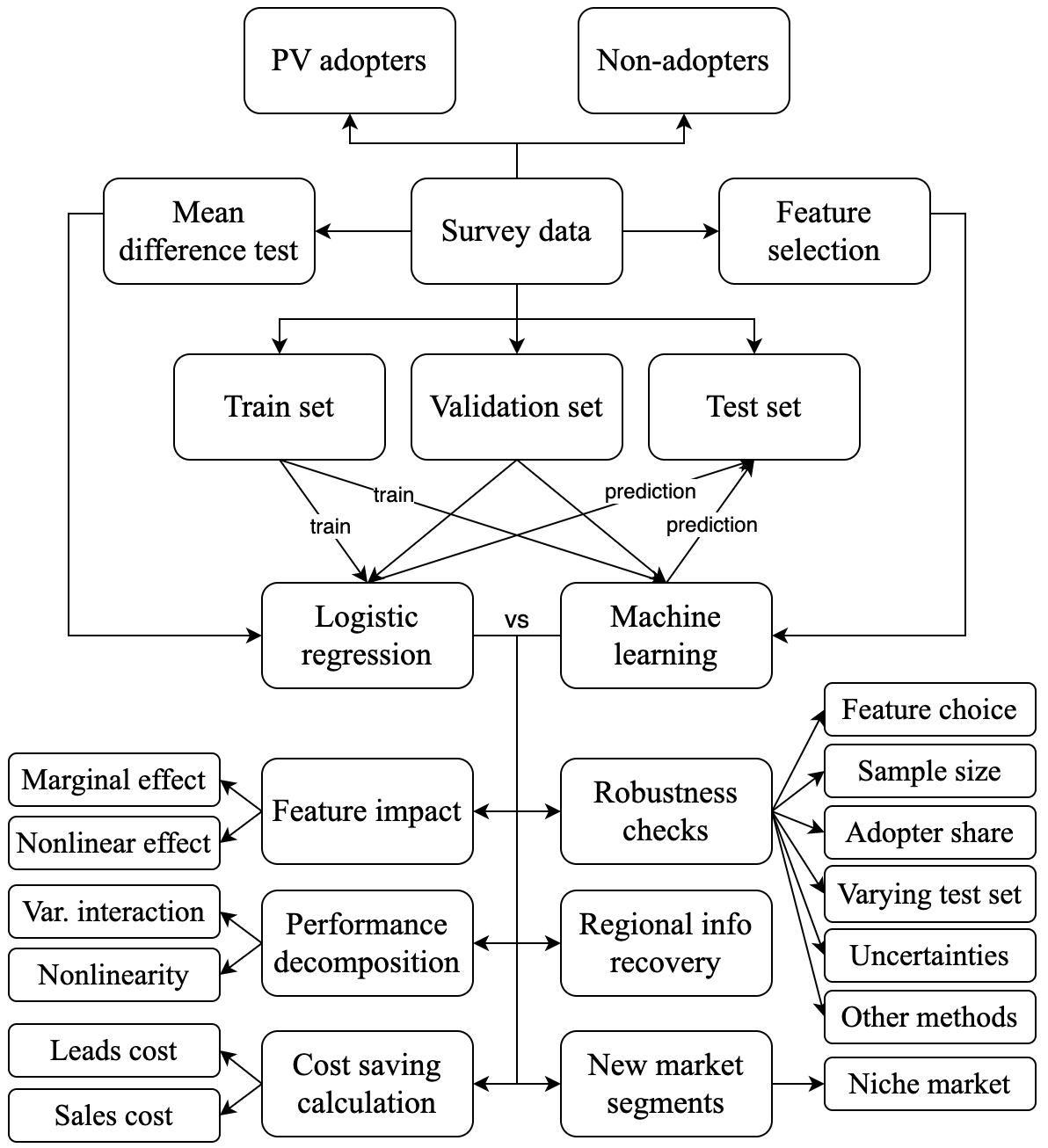
Fig. S9.** Flow chart of overall research methodology in the paper.

**Fig. S10.** Block permutation test results with original data and with resampled data for summer bill and house owner’s age. The resampled data are to derive very similar empirical distributions from the original distinctive distributions. The cost of doing so is that the sample size becomes relatively smaller, since we did not use sampling with replacement to manually increase our sample size.

**Table S1.** Summary of PV adoption literature with samples from both adopters and non-adopters at the household level. Typical sample size is a few hundreds, and a few studies have a sample size over 1,000 (one of these studies comes from one of our co-authors). Literature with data only on either PV adopters or non-adopters is not included here. Adoption studies on other solar technologies than PV are excluded, too.

| Paper | Region | Sample | Method | Factor | Journal |
| --- | --- | --- | --- | --- | --- |
| Balcombe et al. (2014) | UK | *N*=291: 113 adopters, 87 considerers, 91 rejecters | Hierarchical Bayes | Financial factors, energy independence, price hedging, environmental concerns, information barriers | Applied Energy |
| Sigrin et al. (2015) | US | *N*=2024: 1234 adopters and 790 non-adopters | T-test | Education, income, home size, home value, environmental concerns | Environmental Research Letters |
| Vasseur and Kemp (2015) | Netherlands | *N*=817: 38 adopters and 779 non-adopters | Logit | Perceived relative advantage, complexity, social influence, and knowledge | Renewable and Sustainable Energy Reviews |
| Fleiß et al. (2017) | Austria | *N*=533: 152 adopters and 381 non-adopters | Logit | Financial beliefs, age, gender, education; environmental concerns | Journal of Cleaner Production |
| Bashiri and Alizadeh (2018) | Iran | *N*=345: 259 adopters and 86 non-adopters | Logit | Family size, income, education, house ownership, number of residential units, environmental problems, innovation and knowledge | Renewable and Sustainable Energy Reviews |
| Guta (2018) | Ethopia | *N*=195: 107 adopters and 88 non-adopters | Logit | Wealth, education, gender, age, household size, land area | Journal of Cleaner Production |
| Bao et al. (2020) | US | *N*=1773: 563 adopters and 1210 non-adopters | Hierarchical Bayes | Reviewer rating, collaboration style, technology, project time, warranty, savings, income, age | Renewable Energy |
| Mundaca and Samahita (2020) | Sweden | *N*=208: All potential adopters; but with an analysis of 161 adopters in the Appendix | Ordered logit | Visibility, peer effect, subsidy, size, environmentalism, income, age, gender, nationality | Energy Research and Social Science |
| Irfan et al. (2021) | India | *N*=1551: 297 PV adopters and 1254 non-adopters | Probit | Entrepreneurship, income, age, sex, family size, religion, social group, occupation, place of residence | Technological Forecasting & Social Change |
| Alipour et al.  (2022) | Australia | *N*=604: 360 PV adopters and 244 non-adopters | Mean difference | Age, gender, education, income, household size, household age, etc. | Renewable Energy |
| Moglia et al. (2022) | Australia | *N*=1042: 23% PV owners, 15% with an intention | Logit | Dwelling type, tenure, # bedrooms, roof space, various PV beliefs | Journal of Artificial Societies and Social Simulation |
| Zeru and Guta (2022) | Ethopia | *N*=371: 228 PV adopters and 143 non-adopters | Logit | Income, land, cattle, gender, age, family size, marriage, education, house type, prior information, training, credit, phone, media, etc. | Working paper |

**Table S2.** Sample size distribution in four studied states. Percentages of the total sample size shown in parentheses.

| Type | Arizona | California | New Jersey | New York | Total |
| --- | --- | --- | --- | --- | --- |
| Adopter | 109 (3%) | 1176 (33%) | 185 (5%) | 186 (5%) | 1656 (46%) |
| Non-adopter | 455 (13%) | 522 (15%) | 421 (12%) | 516 (14%) | 1914 (54%) |
| Total | 564 (16%) | 1698 (48%) | 606 (17%) | 702 (19%) | 3570 (100%) |

**Table S3.** Summary statistics (*N*=3,570) of various household attributes used in the paper. Categorical variables are shown numerically, including adopter, education, square footage, kids at home, retired, and party affiliation.

| Variable | Description | Mean | St.d | Min | Max |
| --- | --- | --- | --- | --- | --- |
| Adopter | Adoption status (0: Non-adopters; 1: Adopters) | 0.46 | 0.50 | 0 | 1 |
| Summer bill | Summer monthly electricity bill pre-PV | 217 | 146.1 | 15 | 1000 |
| Winter bill | Winter monthly electricity bill pre-PV | 164 | 115.8 | 15 | 1000 |
| Capacity factor | Ratio of actual electricity generation output divided by theoretical maximum over a period of time (%) | 16.8 | 2.21 | 12.4 | 20.3 |
| Income | Household income levels (in $1,000) | 109 | 67.4 | 17.5 | 350 |
| Education | Homeowner’s education (1: High school or less; 2: Some college; 3: Bachelor; 4. Graduate) | 2.66 | 1 | 1 | 4 |
| Square footage | House square footage (1: <1,000; 2: 1,000-1,500; 3: 1,500-2,000; 4: 2,000-3,000; 5: 3,000-4,000; 6: >4,000) | 3.25 | 1.07 | 1 | 6 |
| Kids at home | Having kids at home or not (0: No; 1: Yes) | 0.29 | 0.46 | 0 | 1 |
| # people | # of people living in the house | 2.2 | 0.79 | 1 | 4 |
| Age | Age of homeowner | 54.9 | 14.2 | 18 | 94 |
| Retire | Homeowner retired or not (0: No; 1: Yes) | 0.34 | 0.47 | 0 | 1 |
| Party affiliation | Party affiliation of homeowner (1: Democrat; 2: Republican; 3: Independent) | 2.06 | 0.83 | 1 | 3 |

**Table S4.** Distributional statistics, variable transformation and predictive performance. Only summer bill and income are Box-Cox transformed.

| Statistics | Summer bill | | | Income | | | Winter bill | |
| --- | --- | --- | --- | --- | --- | --- | --- | --- |
|  | Raw | Logged | Box-Cox | Raw | Logged | Box-Cox | Raw | Logged |
| Skewness | 1.59 | -0.41 | -0.005 | 1.46 | -0.36 | 0.0005 | 2.52 | -0.07 |
| Kurtosis | 6.87 | 3.21 | 2.90 | 5.61 | 3.22 | 3.03 | 14.23 | 3.12 |
| Prediction | Sensitivity | | | Specificity | | |  |  |
|  | Raw | Logged | Box-Cox | Raw | Logged | Box-Cox |  |  |
| Logistic | 63.7% | 66.5% | 65.6% | 74.3% | 74.9% | 75.1% |  |  |
| XGBoost | 87.0% | 87.0% | 87.0% | 87.7% | 87.7% | 87.7% |  |  |

**Table S5.** Correlation table for continuous variables (*N*=3,570). Since the highest correlation among these variables is 0.55, multicollinearity is not a serious issue here.

|  | Summer bill | Winter bill | Capacity factor | Income | # peo | Age |
| --- | --- | --- | --- | --- | --- | --- |
| Summer bill | 1 | 0.55 | 0.11 | 0.27 | 0.19 | -0.06 |
| Winter bill |  | 1 | -0.09 | 0.24 | 0.18 | -0.05 |
| Capacity factor |  |  | 1 | 0.00 | -0.03 | 0.09 |
| Income |  |  |  | 1 | 0.12 | -0.08 |
| # peo |  |  |  |  | 1 | -0.11 |
| Age |  |  |  |  |  | 1 |

**Table S6.** Block permutation tests for household features between PV adopters and non-adopters. Variable description and summary statistics are shown in Supplementary Table 2. The block permutation test uses state and county as the block variable, respectively, to better meet the exchangeability assumption of the observations. We use state as our block variable for two reasons: 1) There are potential natural differences among four states, such as meteorology, and 2) many socio-demographic variables including household electricity bills and family income could vary from one state to other. Thus, it is likely that different states have different distributions of these variables, violating the exchangeability assumption of observations across states (Block permutation test does not require this assumption, though). Considering that some states in our dataset, such as California, span long north to south distances creating within-state variation in factors such as insolation, we have further reduced our block size to a county. Lastly, we draw a common distribution from four states to force them to have similar distributions and better conduct the permutation test, and results are very similar (see Fig. S10). ***P < 0.01, **P < 0.05, *P < 0.10.

| Variable | Permutation test | Block (state) permutation test | Block (county) permutation test | Prediction  rate |
| --- | --- | --- | --- | --- |
| Summer bill | 16.0*** | 14.6*** | 12.0*** | 0.62 |
| Winter bill | 10.3*** | 8.7*** | 8.0*** | 0.61 |
| Capacity factor | 13.6*** | 4.7** | 3.6*** | 0.67 |
| Income (in $1,000) | 9.7*** | 6.6*** | 6.4*** | 0.58 |
| # people | 7.5*** | 5.7*** | 4.4*** | 0.55 |
| Age | 8.4*** | 7.2*** | 5.5*** | 0.57 |
| Education | 8.9*** | 7.3*** | 6.2*** | 0.55 |
| Square footage | 5.9*** | 4.2*** | 3.5*** | 0.56 |
| Kids at home | 1.4 | 1.1 | 0.1 | 0.51 |
| Retired | 3.7*** | 3.8*** | 2.4** | 0.53 |
| Party affiliation | 2.8** | 0.7 | 0.5 | 0.52 |

**Table S7.** Model fitting (*N*=2,857) and out-of-sample prediction (*N*=713) results in logistic regression (logit) and XGBoost with different explanatory variable sets and model specifications. Column (2) is the baseline model specification used in the paper, which overall exhibits best out-of-sample prediction results than others. County-clustered standard errors are used for all logistic regressions. ***P < 0.01, **P < 0.05, *P < 0.10.

| Logit |  | (1) | (2) | (3) | (4) |
| --- | --- | --- | --- | --- | --- |
|  | Summer bill (logged) | 1.85*** | 1.84*** | 1.66** | 1.76*** |
|  |  | (0.32) | (0.32) | (0.30) | (0.30) |
|  | Winter bill (logged) | 1.71*** | 1.70*** | 1.52*** | 1.81*** |
|  |  | (0.27) | (0.27) | (0.24) | (0.29) |
|  | Capacity factor (%) | 1.27*** | 1.26*** | 1.25*** | 1.26*** |
|  |  | (0.10) | (0.10) | (0.10) | (0.10) |
|  | Income (logged) | 1.72*** | 1.72*** | 1.41*** | 1.67*** |
|  |  | (0.14) | (0.15) | (0.11) | (0.14) |
|  | Some college | 1.82*** | 1.82*** | 1.83*** | 1.89*** |
|  |  | (0.26) | (0.26) | (0.24) | (0.27) |
|  | Bachelor | 2.47*** | 2.46*** | 2.28*** | 2.35*** |
|  |  | (0.34) | (0.33) | (0.29) | (0.31) |
|  | Graduate | 2.24*** | 2.24*** | 2.03*** | 2.23*** |
|  |  | (0.34) | (0.34) | (0.28) | (0.33) |
|  | Sqft (1000,1500] | 1.16 | 1.17 |  | 1.333 |
|  |  | (0.29) | (0.30) |  | (0.36) |
|  | Sqft (1500,2000] | 1.22 | 1.23 |  | 1.57* |
|  |  | (0.29) | (0.29) |  | (0.41) |
|  | Sqft (2000,3000] | 0.69 | 0.70 |  | 0.91 |
|  |  | (0.18) | (0.18) |  | (0.26) |
|  | Sqft (3000,4000] | 0.29*** | 0.30*** |  | 0.44*** |
|  |  | (0.08) | (0.08) |  | (0.13) |
|  | Sqft (4000+) | 0.13*** | 0.13*** |  | 0.16*** |
|  |  | (0.05) | (0.05) |  | (0.07) |
|  | Kids at home | 1.70*** | 1.69*** | 1.65*** |  |
|  |  | (0.22) | (0.22) | (0.22) |  |
|  | # people | 1.28*** | 1.29*** | 1.27*** |  |
|  |  | (0.09) | (0.09) | (0.09) |  |
|  | Age | 1.04*** | 1.04*** | 1.04*** |  |
|  |  | (0.00) | (0.00) | (0.00) |  |
|  | Retired | 0.95 |  |  |  |
|  |  | (0.09) |  |  |  |
|  | Republican | 0.99 |  |  |  |
|  |  | (0.13) |  |  |  |
|  | Independent | 1.13 |  |  |  |
|  |  | (0.13) |  |  |  |
|  | Log likelihood | -1,644 | -1,645 | -1,700 | -1,704 |
|  | A.I.C. | 3,326 | 3,323 | 3,421 | 3,434 |
| In-sample prediction |  |  |  |  |  |
|  | Overall accuracy | 0.71 | 0.71 | 0.68 | 0.69 |
|  | Sensitivity | 0.67 | 0.67 | 0.65 | 0.64 |
|  | Specificity | 0.74 | 0.74 | 0.72 | 0.73 |
|  | AUC score | 0.77 | 0.77 | 0.74 | 0.74 |
| Out-of-sample prediction |  |  |  |  |  |
|  | Overall accuracy | 0.71 | 0.71 | 0.69 | 0.69 |
|  | Sensitivity | 0.67 | 0.66 | 0.67 | 0.61 |
|  | Specificity | 0.75 | 0.75 | 0.73 | 0.75 |
|  | AUC score | 0.77 | 0.77 | 0.77 | 0.75 |
| XGBoost |  | (1) | (2) | (3) | (4) |
| In-sample prediction |  |  |  |  |  |
|  | Overall accuracy | 0.89 | 0.90 | 0.90 | 0.87 |
|  | Sensitivity | 0.90 | 0.92 | 0.92 | 0.89 |
|  | Specificity | 0.88 | 0.89 | 0.88 | 0.86 |
|  | AUC score | 0.96 | 0.97 | 0.97 | 0.95 |
| Out-of-sample prediction |  |  |  |  |  |
|  | Overall accuracy | 0.86 | 0.87 | 0.87 | 0.85 |
|  | Sensitivity | 0.86 | 0.87 | 0.87 | 0.84 |
|  | Specificity | 0.86 | 0.88 | 0.87 | 0.86 |
|  | AUC score | 0.92 | 0.93 | 0.93 | 0.92 |

**Table S8.** Post-estimation diagnosis results for logistic regression. Two versions of variance inflation factor (VIF) values for multicollinearity tests are shown below: First is for all numeric and dummy-coded variables used in our baseline model, and second is for the same group of variables but with a slightly different set of dummy-coded variables for square footage (‘sqft’). This new set of dummy-coded variables for square footage uses a different reference category: (1500,2000] instead of (0,1000]. The intention is to show that the high VIF values for dummy-coded variables can be simply reduced by changing the reference category and thus can be safely ignored [68,69]. As to the tests of outlier, the maximum Cook’s distance is less than 0.01, and no standardized residuals have an absolute value greater than 3. Lastly, the problem of heteroscedasticity is automatically dealt with via robust standard errors. cf, capacity factor.

| V1 | ln_sum | ln_win | cf | ln_inc | edu2 | edu3 | edu4 | sqft2 |
| --- | --- | --- | --- | --- | --- | --- | --- | --- |
| VIF | 1.46 | 1.48 | 1.12 | 1.29 | 2.66 | 2.61 | 2.66 | 4.92 |
|  | sqft3 | sqft4 | sqft5 | sqft6 | kids | n_peo | age |  |
| VIF | 6.59 | 6.61 | 2.82 | 1.56 | 1.51 | 1.08 | 1.55 |  |
|  |  |  |  |  |  |  |  |  |
| V2 | ln_sum | ln_win | cf | ln_inc | edu2 | edu3 | edu4 | sqft1 |
| VIF | 1.46 | 1.48 | 1.12 | 1.29 | 2.66 | 2.61 | 2.66 | 1.10 |
|  | sqft2 | sqft4 | sqft5 | sqft6 | kids | n_peo | age |  |
| VIF | 1.28 | 1.40 | 1.24 | 1.12 | 1.51 | 1.08 | 1.55 |  |

**Table S9.**  Sensitivity analysis on the train-test split. The results show that using either 70% or 90% of the data as the training set does not change much the predictive performances of the two baseline models.

| 70% training | Model | Accuracy | Sensitivity | Specificity |
| --- | --- | --- | --- | --- |
|  | Logistic | 0.71 | 0.66 | 0.75 |
|  | XGBoost | 0.85 | 0.87 | 0.84 |
| 80% training | Model | Accuracy | Sensitivity | Specificity |
|  | Logistic | 0.71 | 0.66 | 0.75 |
|  | XGBoost | 0.87 | 0.87 | 0.88 |
| 90% training | Model | Accuracy | Sensitivity | Specificity |
|  | Logistic | 0.72 | 0.70 | 0.74 |
|  | XGBoost | 0.87 | 0.85 | 0.88 |

**Table S10.** Sensitivity analysis on using three states as training data and the fourth state as test data. The results in Panel A show that all models produce very similar results for all testing states except California, in which the training dataset is the smallest and the testing dataset is the largest (48% of the sample size). This suggests that one can at least pool the data from Arizona, New Jersey and New York together. For these three states, the better prediction performance for XGBoost is apparent. Panel B runs the baseline models for California only, and the better performance for XGBoost is also apparent. In all models below, capacity factor is excluded in order not to emphasize the between-state differences.

| Panel A:  3 tests 1 | Test state | Prediction Accuracy | | | |
| --- | --- | --- | --- | --- | --- |
|  |  | Logistic | | XGBoost | |
|  | AZ | 0.61 | | 0.84 | |
|  | CA | 0.42 | | 0.65 | |
|  | NJ | 0.60 | | 0.81 | |
|  | NY | 0.64 | | 0.78 | |
| Panel B:  CA | Model | Accuracy | Sensitivity | | Specificity |
|  | Logistic | 0.77 | 0.90 | | 0.47 |
|  | XGBoost | 0.83 | 0.92 | | 0.63 |

**Table S11.** Comparing logistic regression and XGBoost with other classification models, with and without state dummy variables. As our dataset includes four different states, including state dummy variables into the model should remove any between-state variation. The bottom panel shows that including state dummy variables improve the prediction performance for logistic regression, linear discriminant analysis (LDA), quadratic discriminant analysis (QDA), and support vector machine (SVM), but it does not improve the performance for random forest and XGBoost. Furthermore, the dominant performance of machine learning is apparent. The similar results for XGBoost before and after including state dummy variables suggest that machine learning is able to discover key latent information (such as geography) underneath the data.

| Without state |  |  |  |  |
| --- | --- | --- | --- | --- |
|  | Model | Accuracy | Sensitivity | Specificity |
|  | Logistic | 0.71 | 0.66 | 0.75 |
|  | LDA | 0.71 | 0.67 | 0.75 |
|  | QDA | 0.69 | 0.81 | 0.58 |
|  | SVM | 0.72 | 0.64 | 0.80 |
|  | Random forest | 0.82 | 0.77 | 0.86 |
|  | XGBoost | 0.87 | 0.87 | 0.88 |
| With state |  |  |  |  |
|  | Model | Accuracy | Sensitivity | Specificity |
|  | Logistic | 0.76 | 0.70 | 0.82 |
|  | LDA | 0.76 | 0.70 | 0.82 |
|  | QDA | 0.75 | 0.76 | 0.74 |
|  | SVM | 0.76 | 0.69 | 0.83 |
|  | Random forest | 0.83 | 0.79 | 0.87 |
|  | XGBoost | 0.87 | 0.86 | 0.87 |

**Table S12.**  Scenario definition for XGBoost model runs. Colsample is the max fraction of columns to be randomly drawn in each decision tree, round is the max number of decision trees to be used in model-fitting, and depth is the max tree depth or tree layers to be used. Colsample and round together determine the degree of variable interaction in growing decision trees, whereas depth determines the degree of nonlinearity for a single variable in decision trees.

| Run | Colsample | Round | Depth |
| --- | --- | --- | --- |
| s1 | 1 | 1000 | 10 |
| s2 | 0.07 | 500 | 10 |
| s3 | 0.07 | 100 | 10 |
| s4 | 0.07 | 50 | 10 |
| s5 | 0.07 | 30 | 10 |
| s6 | 0.07 | 20 | 10 |
| s7 | 0.07 | 20 | 8 |
| s8 | 0.07 | 20 | 6 |
| s9 | 0.07 | 20 | 5 |
| s10 | 0.07 | 20 | 4 |

**Table S13.** Sensitivity analysis on predicting adopters from low-income households. We randomly draw all the testing cases from below-average income households (704 out of 1,967, where 704 is 20% of the overall sample size). The results show that while logistic regression improves its performance of predicting non-adopters (i.e., specificity) to some extent, its performance of predicting adopters (i.e., sensitivity) worsens. However, XGBoost takes a more balanced view. The better performance of XGBoost in predicting PV adopters successfully is even more apparent in this case. The overall predictive power of XGBoost stays roughly the same.

| Model | 20% from all samples | | | 20% from below-average income | | |
| --- | --- | --- | --- | --- | --- | --- |
|  | Accuracy | Sensitivity | Specificity | Accuracy | Sensitivity | Specificity |
| Logistic | 0.71 | 0.66 | 0.75 | 0.71 | 0.59 | 0.78 |
| XGBoost | 0.87 | 0.87 | 0.88 | 0.88 | 0.86 | 0.90 |

**Table S14.**  A random sample (*N*=30) of the dataset used in the paper.


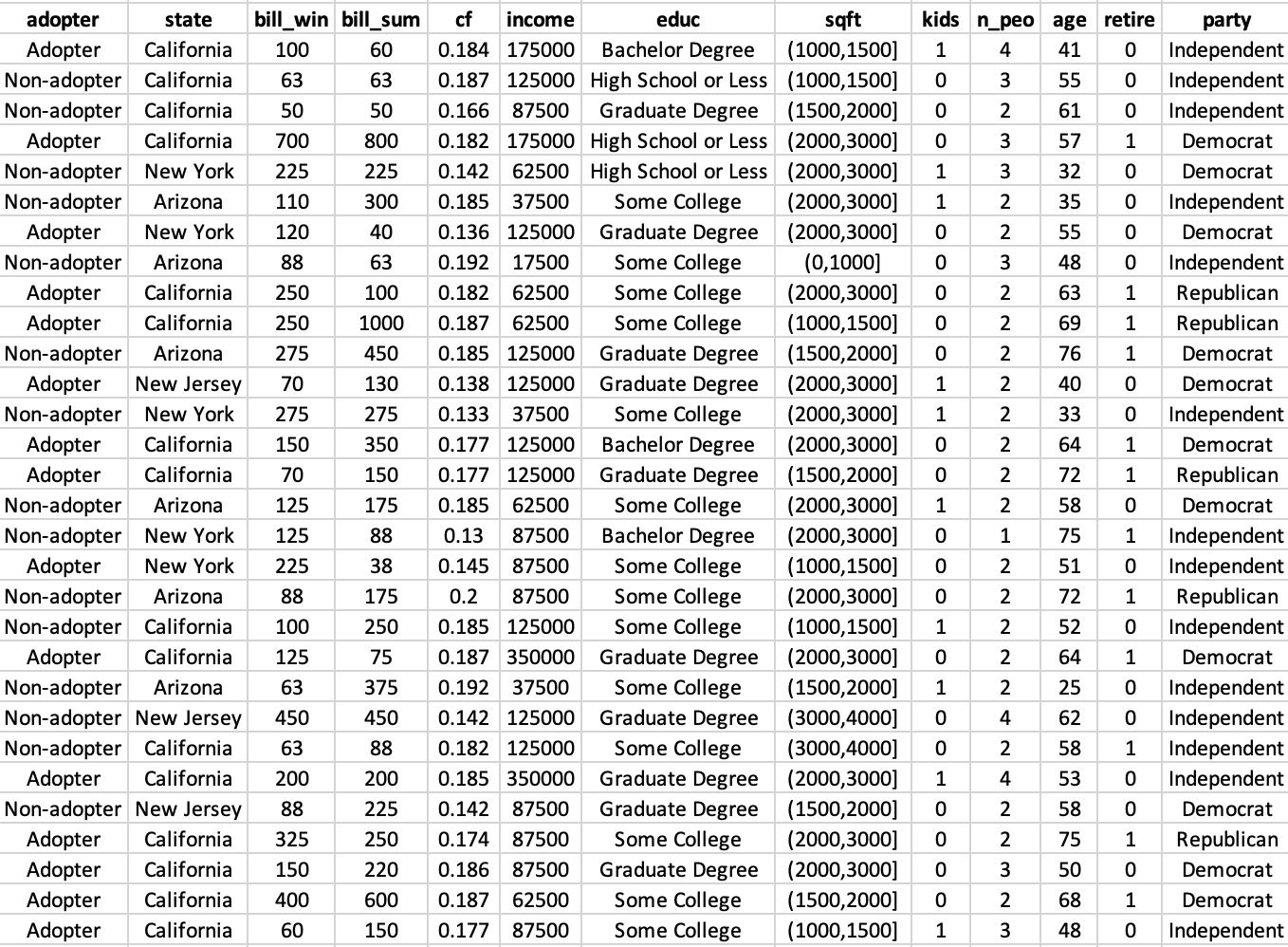


**SI References:**

1. B. Sigrin, J. Pless, E. Drury, Diffusion into new markets: evolving customer segments in the solar photovoltaics market. *Environ. Res. Lett.* **10**, 084001 (2015).

2. S. A. Robinson, V. Rai, Determinants of spatio-temporal patterns of energy technology adoption: An agent-based modeling approach. *Applied Energy* **151**, 273–284 (2015).

3. M. Moezzi, A. Ingle, L. Lutzenhiser, B. O. Sigrin, “A Non-Modeling Exploration of Residential Solar Photovoltaic (PV) Adoption and Non-Adoption” (National Renewable Energy Lab. (NREL), Golden, CO (United States), 2017) https:/doi.org/10.2172/1379469 (April 28, 2021).

4. L. Murray, H. Nguyen, Y.-F. Lee, M. Remmenga, D. Smith, Variance inflation factors in regression models with dummy variables in *Annual Conference on Applied Statistics in Agriculture*, (New Prairie Press, Kansas State University Libraries, 2012), pp. 161–177.

5. B. Güneralp, *et al.*, Global scenarios of urban density and its impacts on building energy use through 2050. *PNAS* **114**, 8945–8950 (2017).
